# An Integrated Conceptual Framework of Behavioral Intervention Technologies to Promote Healthcare Utilization Among Socially-Marginalized Populations in the United States

**Published:** 2022-05-06

**Authors:** Chen Zhang, Shelby Przybylek, Amy Braksmajer, Yu Liu

**Affiliations:** 1 School of Nursing, University of Rochester Medical Center, Rochester, NY, USA; 2 Sociology Department, The State University of New York at Geneseo, Geneseo, NY, USA; 3 Department of Public Health Science, University of Rochester Medical Center, Rochester, NY, USA

**Keywords:** Behavioral intervention technologies, Theoretical framework, Vulnerable populations, Health disparities

## Abstract

In the U.S., socially marginalized groups disproportionately shoulder the burden of health outcomes. The evolving development of behavioral intervention technologies provides opportunities to support users in changing behaviors and improving health. No conceptual model is available to guide technology-based behavioral interventions among vulnerable groups in the new era of digitalization. Our conceptual framework combines the Behavioral Model of Healthcare Utilization (HCU) for Vulnerable Populations and the Integrated Model of Behavioral Prediction (IMBP). With the Socioecological Model leading the vertical direction, the overarching HCU-IBMP model is incorporated by the Behavioral Intervention Technology-Technological Instantiation Framework (BIT-Tech). The HCU model explains how vulnerable populations influence healthcare access and utilization behaviors by personal and contextual factors. Through the lens of the IMBP, healthcare utilization intention is highly predictable and maybe even causally correlated with the corresponding behavior. To assist the healthcare utilization intention translates into the corresponding behaviors, we employ a medium using the technological implementation in a system that can assist the end-users in adopting the desired behavior. With the integrated model that combines a technological framework with the behavioral components, the BIT-Tech guides the procedure intervention development. Our integrated conceptual framework indicated that theoretical and technical components should be considered during a technological tool development to support the research community. A real-world intervention design has been demonstrated using the framework to guide behavioral intervention technologies to promote PrEP care and utilization among racial/ethnic minority women in the U.S.

## Introduction

The burden of healthcare in the United States (U.S.) disproportionately affects groups that have been economically and socially marginalized. These individuals include economically disadvantaged individuals, racial and ethnic minorities, immigrants, rural residents, or those with chronic health conditions, such as HIV [1]. These individuals usually have higher mortality and morbidity but receive lower-quality care than general populations [2]. For instance, both incidence and prevalence of HIV/AIDS are highest among racial and ethnic minorities, and people with low socioeconomic status are less likely to have linkage to care or retain in care [3]. Similarly, the prevalence of two or more common chronic health conditions among Black individuals was much higher than their White peers [2]. Researchers observed the same pattern across different timespans, such as 1999 to 2000 compared to 2009 to 2010, and different age groups, such as 45–64 years of age versus 65+ years of age [2].

Despite efforts made to reduce disparities in healthcare in the U.S., groups that have been economically and socially marginalized continue to have the risk of disparate healthcare access, utilization, and outcomes due to economic, cultural, language, and geographical barriers embedded within their life context [1,3,4]. At the institutional and societal level, obstacles include shortage of healthcare providers, lack of navigation within the complex medical care system, medical mistrust or biased healthcare environment, or lack of insurance coverage [5,6]. At the interpersonal and intrapersonal level, barriers include limited health literacy, language issues, lack of transportation, negative experience from past healthcare experiences, and other competing priorities, such as shelter, food, substance use, and mental health [5,6]. All previously mentioned factors may contribute to delayed care and poor health outcomes among groups that have been economically and socially marginalized [1,3,4]. Therefore, we call for scalable, sustainable, accessible, and cost-effective strategies to address these obstacles among disproportionately affected groups who desperately need improved healthcare utilization and health outcomes.

In the new era of digitalization and the evolving technology development, behavioral intervention technologies (BIT) have been successfully helping patients with risk assessment and disease management for various conditions [7–9]. For instance, Foley et al. [10] have successfully recruited a group of socioeconomically disadvantaged racial/minority individuals in a digital health obesity treatment intervention. The same research group also found mobile-based text-messaging adherence interventions indicating a more significant weight loss among a group of racial and ethnic minority women [11]. Similarly, a review focusing on technology-based interventions targeting mental health status among HIV/AIDS individuals revealed that BIT might effectively serve as a vehicle to address prevalent health disparities among groups placed at increased risk of HIV [9].

However, there is a lack of a conceptual model available to guide the Development of BIT among vulnerable populations. A few gaps have been identified in the existing literature: a lack of combination between behavioral theories and technical components. Firstly, some studies employed behavioral theories as the theoretical framework while describing technical components separately [10,12]. Secondly, there is a lack of measurements for both behavioral and usage outcomes or a lack of time-varied measurements, including short-, medium-, and long-term measurements [9,12]. For instance, most existing studies used technology-based interventions only focused on behavioral changes, such as smoking cessation and HIV testing uptake, but rarely do any of them measure usage outcomes, including engagement or acceptance of the used technology [13]. Thirdly, there is a lack of diversities in technologies. Existing studies have focused on text-messaging or mobile-health behavioral interventions [7,8,13]. They have several identified barriers: not being available 24/7, no prompt responses, and high cost [7,8,13]. These barriers may hinder the implementation and generalization of the employed technologies [13]. With the Development of digitalization, more advanced strategies that agree with those identified barriers need to be acknowledged and employed for behavioral interventions.

Documented BIT include sensor-trackers (e.g., heart rate, step count), artificial intelligence (A.I.)-powered Chatbot (an automated conversational agent), and momentary ecological assessment (which can repeatedly assess subjects’ behaviors and experience them in real-time) [14,15]. To fill these gaps, we call for a theoretical framework to combine behavioral and technical components to guide intervention development while assessing behavioral and usage outcomes to guide the translation from technology into health research to improve health outcomes among disproportionately affected groups.

## The General Model of the Hybrid Framework

The hybrid conceptual framework combines Gelberg-Andersen’s Behavioral Model of Healthcare Utilization (HCU) for Vulnerable Populations [16] and the Integrated Model of Behavioral Prediction (IMBP) [17,18]. The Socioecological Model [19] is leading the vertical direction; the overarching combined HCU-IBMP model is incorporated by the Behavioral Intervention Technology (BIT)-Technological Instantiation (BIT-Tech) Framework [20].

Gelberg-Andersen’s model explains how personal and contextual factors among vulnerable populations collectively influence healthcare access and utilization behaviors [16]. Three categories of the corresponding determinants include: (1) factors that predispose individuals favorably or unfavorably towards a given behavior, including both individual and social-structure level determinants; (2) factors associated with both perceived needs and objective appraisals for health-seeking behaviors or medical regimen adherence. For instance, based upon this framework, “perceived HIV risk” is considered as “perceived needs,” and “actual HIV risk” is the “objective appraisals”; and (3) factors embedded within personal, familial, community, and institutional level that either enable or impede the healthcare utilization behavior. Through the lens of the behavioral prediction components (from the IMBP), healthcare utilization intention is highly predictable and maybe even causally correlated with the corresponding behavior [17,21].

The desired behavior is a joint function of attitudes about performing the behavior, perceived norms regulating the performance of the intended behavior, and self-efficacy, or the individual’s perception of his/her ability to perform the behavior [18]. These key factors interact with background determinants and population characteristics, such as needs, predisposing, and enabling factors. With the guidance of the socioecological model, the framework indicates that factors embedded at different levels work vertically towards healthcare utilization behaviors.

We know that a technological delivery framework that addresses the above-mentioned behavioral components is essential to assist the healthcare utilization intention, as it translates into corresponding behaviors. We employ a medium using the technological implementation in a system that can assist the end-users in adopting the desired behavior. Specifically, the “end-users” in the current model refer to “vulnerable groups” who may benefit from the application of the BIT, and the “desired behavior” refers to the “use of health services to prevent or cure health problems (a.k.a., health utilization)” in the healthcare scenario.

We use alphabetical notations to illustrate a few key components in this hybrid model. Data include information collected from an end-user [Dt(U)] and from the environment where the end-user interacts with [Dt(E)] at a time t. Intervention repositories (It) store all developed intervention elements and strategies that aim to improve the desired behaviors and can be implemented at time t. Workflow (Wt) defines when and under what conditions intervention components will be delivered or implemented at time t. With the assistance of the BIT, the hybrid model aims to attain both clinical/behavioral (At) and usage outcomes (A’t) at the time t. Clinical/behavioral outcomes (At) refer to changes in behaviors, knowledge, skills, or motivation for health-related behaviors, and usage outcomes (A’t) focus on accepting and engaging with the BIT. With the hybrid model that integrates a technological framework and the behavioral components, the BIT-Tech guides the procedure by collecting data [Dt=Dt(U)+Dt(E)] from both individuals [Dt(U)] and environment [Dt(E)], selecting tools from the intervention repositories (It), tailoring elements, characteristics and workflow (Wt) of the intervention to end-users, and achieving both behavioral/clinical (At) and usage outcomes (A’t).

Our integrated conceptual framework includes both theoretical and technological components; it aims to achieve the ultimate goals of both usage and clinical/behavioral outcomes. The usage outcomes include acceptance, feasibility, and engagement of the BIT tools, while the clinical and behavioral outcomes include improved healthcare utilization and reduced disease incidence and prevalence.

## Application of the Hybrid Framework

As a case study using critical components from the hybrid framework, we will describe the context-specific application for planning an intervention to promote Preexposure Prophylaxis (PrEP) care among racial/minority women using an AI-powered Chatbot platform in the U.S.

## Background

HIV continues to impose a heavy burden on women in the U.S. and worldwide. Globally, 17.8 million women aged 15 years and older living with HIV, and 47% of new HIV infections affect females [22]. In the U.S., more than 7,000 women were newly diagnosed with HIV infections in 2018, which accounted for 30% of new infections [23]. Of these, 85% were attributed to heterosexual contact, and 15% were attributed to injection drug use [23]. National representative data indicate a profound health disparity in poor health outcomes and a lack of access to health among racial/ethnic minorities, especially for HIV/AIDS among women in this group [23,24]. Although racial/ethnic women represent 29% of the total women population in the U.S.; they account for 75% of women infected with HIV in 2018 [23,25]. Fewer Black and Hispanic women linked and received HIV medical care after HIV diagnosis. However, more of them experienced higher mortality or comorbidity than their White peers [23,26]. PrEP is efficacious, safe, and cost-effective, but the PrEP used as an HIV prevention strategy is widely underutilized, without tailored and effective interventions.

PrEP trials in women demonstrate that the gender-appropriate implementation of PrEP among high-risk women is feasible and desirable [27–29]. Despite these potential benefits, PrEP uptake among racial/ethnic minority women is deficient. The CDC estimates that 225,000 HIV-negative women in the U.S. have indications for PrEP in 2018. However, only 6.6% of them use PrEP, and only 5.9% and 10.9% of the PrEP-eligible Black and Hispanic women are currently taking it, compared with 42.1% of their White counterparts [26,30]. Moreover, prescription data from the New York State Medicaid program revealed that the proportion of female PrEP users dropped from 45% in 2012 to 22% in 2016 [31]. PrEP care continuum describes a dynamically continuous procedure from identifying an individual at risk for HIV contraction to facilitating, linking and retaining the individual in PrEP care [32]. For women at high risk who would benefit from PrEP, each phase of the continuum represents a potential barrier (and a critical intervention point) to achieving sustained PrEP adherence and protection [32,33]. As the starting point of the PrEP care continuum, low PrEP awareness and lack of information have been frequently cited as one of the primary reasons women with PrEP indications did not use it [33–35]. Research has suggested that once women were informed of PrEP, their willingness to take PrEP increased considerably, especially for Black and Hispanic women [35]. Even among people who are aware of PrEP, “lack of concern about HIV” or “competing priorities” is often cited as a barrier to PrEP uptake [36,37]. The success of engaging racial/minority women in the PrEP care continuum is driven by critical characteristics of the chosen intervention programs [20,38]. Although researchers have employed various strategies to promote the PrEP care continuum among at-risk groups, very few methods are openly available to assist these women with PrEP care [30].

While existing theory-driven programs are widely validated [38–40], there are a few limitations that need to be considered: first, although available interventions are designed to tailor to women’s characteristics, they are not fully producing personally relevant tailored messages or feedback, which has a substantial impact on user’s acceptance, usability, and effectiveness [20,38,41,42]. Second, existing interventions may require high consumption of tangible or intangible resources but have limited generalizability of their usage to a broader audience with a low cost. Moreover, there is a lack of a holistic strategy to target barriers at different levels among women who may be at high risk of HIV infection. Therefore, alternative strategies are needed to expand the current intervention tools while incorporating holism, precision prevention, timeliness, and cost-effectiveness [43,44].

Chatbot technology, using artificial intelligence (AI)-assisted tools including machine learning (ML) and natural language processing (NLP). ML refers to an algorithm that trains a model using provided data to make predictions or decisions. NLP is the ability to recognize, analyze and respond to verbal or written languages. Chatbot has been introduced into the health sector to address current healthcare challenges, such as shortage of healthcare providers and lack of healthcare access [45]. Research has shown positive effects of Chatbot-based interventions [45]. Although the application of Chatbot in HIV prevention services is limited; available studies show promising directions for adopting this tool [46]. For instance, an AI-enabled virtual reality program among HIV-positive men who have sex with men (MSM) showed the potential to facilitate HIV status disclosure [47]. Several AI-led Chatbots launched on public social media, such as Facebook, have shown positive effects and high acceptability in responding to sexual health information for the youths [48–50]. These AI-powered algorithms provide end-users with individually tailored and confidential messages to address their specific needs and requests promptly. However, none of these algorithms have been applied in PrEP care among racial/minority women, in which Chatbot can address identified barriers in a timely, holistically, and cost-effective way. (Table 1).

In this case study, we will employ the Hybrid Framework to guide the intervention to improve PrEP utilization among racial/minority women using the Chatbot technology. In the context-specific Hybrid Framework, the PrEP Care Continuum Framework (PrEP-CC) [32] guides the horizontal direction, and the Socioecological Model [19] leads the vertical direction. Gelberg-Andersen’s model explains how healthcare access and utilization behaviors are collectively influenced by predisposing factors, perceived needs and objective appraisals, and enabling factors. Especially, factors that predispose individuals’ favorably or unfavorably toward a PrEP utilization, including demographics, residential history, living condition (e.g. shelter, stable housing), childhood characteristics (e.g., childhood abuse), substance abuse, and/or societal structure characteristics (e.g., PrEP related stigma); factors associated with both perceived needs (i.e., perceived HIV risks) and objective appraisals (i.e., actual HIV risks) for health-seeking behaviors or medical regimen adherence, as well as their motivation to comply with these referents; and factors embedded within personal (e.g., perceived benefits), familial (e.g., family support), community (e.g., PrEP clinics availability), and institutional level (e.g., medical mistrust, insurance coverages) that either enable or impede the healthcare utilization behavior.

The desired behavior is a joint function of attitudes about performing the behavior, perceived norms that regulate the performance of the intended behavior, and self-efficacy [18]. In this case, attitudes refer to women’s willingness to use PrEP. Perceived norms include “descriptive norms” that refer to “perceptions of what behaviors are typically performed”, and “injunctive norms” that are defined as “perceptions of what behaviors are approved/disapproved by others.” Self-efficacy is the individual’s perception of his/her ability to perform the behavior. These factors interact with background determinants and population characteristics, such as needs, predisposing, and enabling factors. With the guidance of the PrEP-CC, the overarching behavioral model horizontally progresses across each stage along the PrEP care continuum, from PrEP awareness, PrEP use willingness, PrEP use intention, linkage to care, and PrEP uptake, retention, and adherence. Besides, the socioecological model indicates that factors at different levels work vertically towards healthcare utilization behaviors.

To assist the healthcare utilization intention translates into the corresponding behaviors, we employ a BIT tool (i.e., Chatbot platform) that can assist the end-users (i.e., racial/minority women) in adopting the desired behavior (i.e., PrEP utilization) using their desired languages (e.g., English, Spanish). With the guided intervention, we aim to develop a user-centered program to optimize behavioral (i.e., PrEP uptake), clinical outcomes (i.e., reduced HIV risk), and usage outcomes (i.e., acceptance and engagement in Chatbot for PrEP utilization) among racial/minority women.

## Discussion

The Hybrid Framework serves as the conceptual basis for understanding, analyze and design technology-assisted behavioral change interventions among vulnerable populations. It can be used to respond to problems, such as health disparities among vulnerable populations that have not been well-addressed by existing solutions. It helps researchers to identify critical issues and prescribe solutions to research problems. And it facilitates limiting the scope of the constructs and variables to build new knowledge and refine the framework by analyzing and interpreting data and validating theoretical hypotheses [51].

Several strengths of the Hybrid Framework should be acknowledged. First, it is a pioneer work to integrate behavioral theories and technology architecture with specific constructs to guide interventions to improve health equity among people in need. We also used a real-world application to illustrate how researchers can define, design, develop and deploy this framework to reduce health disparities among groups put at increased risk of HIV infections. Researchers can quickly adapt the framework to other groups experiencing disproportionate disease burdens with adjustable components and constructs. However, there are limitations involved that researchers using this framework must keep in mind while using it. The Hybrid Framework may simplify procedures when developing BIT-based programs in real-world settings. It designs as a general framework that should be tailored and modified to meet the needs of target populations when developing a specific BIT program. Furthermore, the Hybrid Framework has not integrated specific procedures while developing the BIT programs. Components such as user-friendly design, sentimental analyses, and automation algorithms should be incorporated when developing their programs [20,51]. As with any other technology, these technologies may be hard to use for less-educated individuals, and the applications of new technologies may further deepen health inequities [52]. In addition, these technologies require constant improvement of information entry to tailor the responses to end-user needs, which may consume more tangible and intangible resources [52]. Last, due to its nature being a newly developed theoretical framework, we have not validated this hybrid framework in any real-world settings [53]. However, our framework provides a network of linked concepts and constructs from validated theories [6,16,18,20,32], and it aims to bring balance to theory and practice in applied scenarios.

Besides, during the BIT-based intervention development, researchers must follow general principles that guide digitalization and A.I. integration within healthcare [54–56], and carefully address health disparities using appropriate machine learning algorithms [57,58]. Key ethical considerations for the current Chatbot platform include minimizing bias, maximizing accuracy, ensuring transparency, and protecting user’s privacy and safety [59,60].

## Conclusion

The hybrid framework builds on several validated theoretical models. It extends the BIT model by applying the Hybrid Framework among groups that have been economically and socially marginalized in specific contexts. The Hybrid Framework incorporates traditional behavioral models with the instantiations of technology-based components. Our framework brings a balance between novelty and continuity. It provides a mind mapping to guide researchers in applying new technologies to traditional behavioral intervention programs for vulnerable populations. For health professionals, including both clinicians and nursing cohorts, integrating technologies into behavioral intervention will be essential to help with groups placed at increased risk or individuals who encounter health disparity or health inequity in health care [61]. We acknowledge that the Hybrid Framework may still be in its infancy. However, we are initiating a necessary conversation to theorize the ongoing reflections on applying digitalization to behavior interventions in our scholarly community.

## Figures and Tables

**Figure 1.**
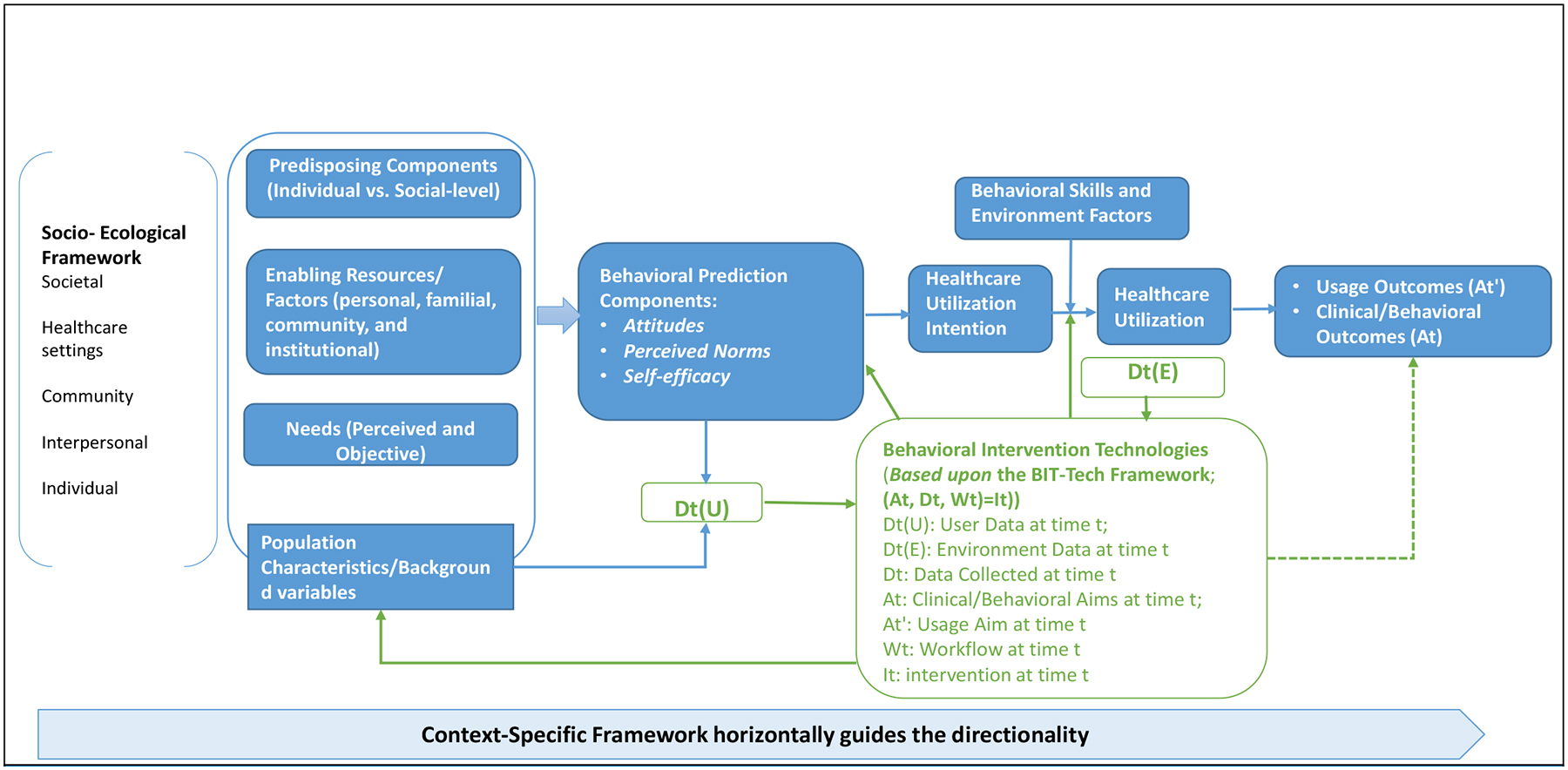
Health Utilization among Vulnerable Populations and Integrated Model of Behavioral Prediction Framework

**Figure 2.**
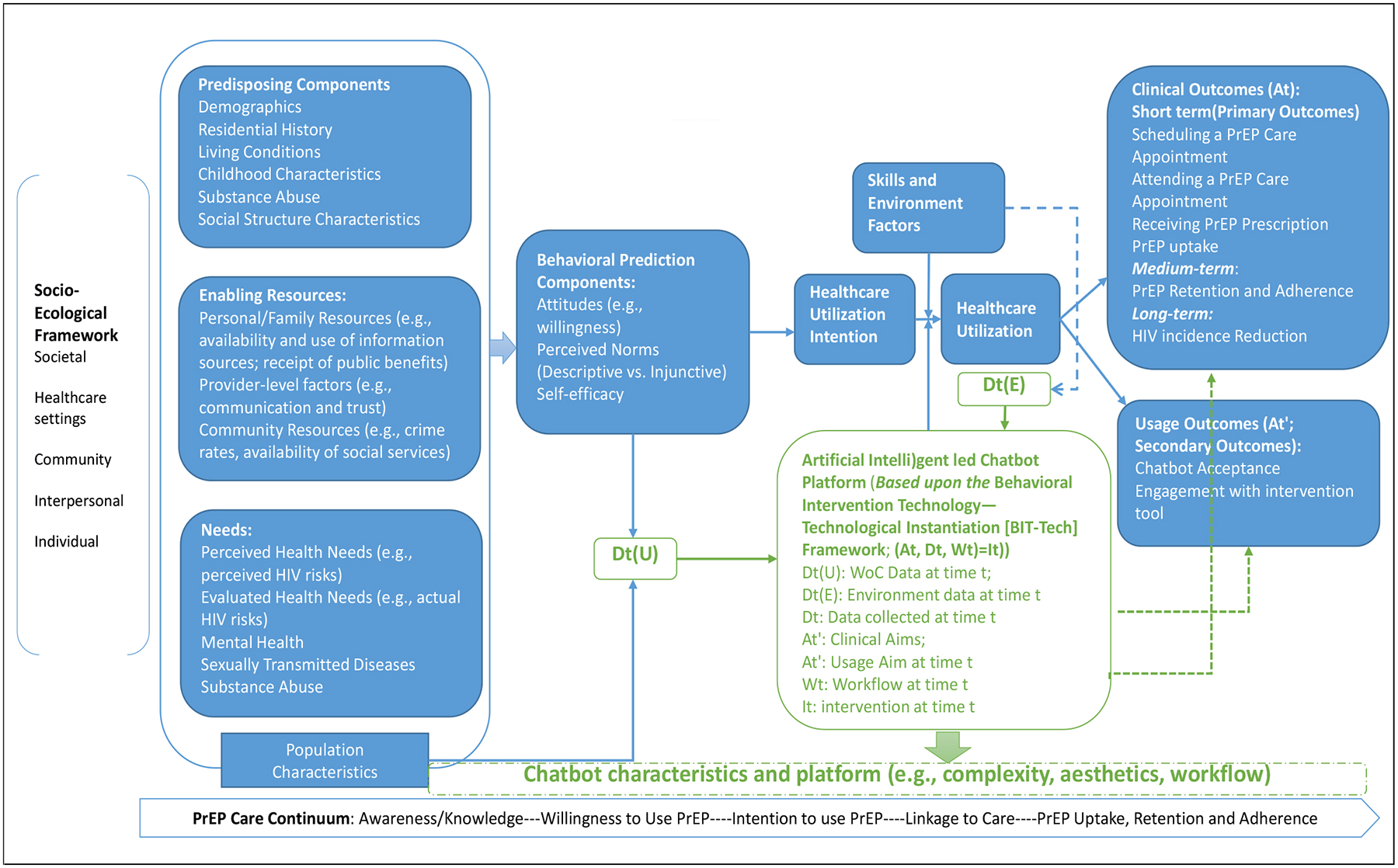
The Hybrid Model of PrEP Care Utilization in Racial/Minority Women

**Table 1:** How Chatbot Addresses Identified Barriers to PrEP care among End-users

Barriers	Chatbot components	Example dialogue (e.g., PrEP navigation)
**Individual-level**		User: hello Chatbot: Hi there! I am a Smartbot, the little robot to assist you. what is your name? Chatbot: PrEP (preexposure prophylaxis) is medicine people at risk for HIV take to prevent getting HIV from sex or injection drug use. There are two medications approved for use as PrEP: Truvada^®^ and Descovy^®^. What do you want to know more about the following topics? Is PrEP Safe? Is PrEP effective? User: Is PrEP safe? Chatbot: PrEP is safe, but some people experience side effects like diarrhea, nausea, headache, fatigue, and stomach pain. These side effects usually go away over time. Do you want to know more about PrEP? Am I eligible for PrEP use? Where can I get PrEP care? ……
Lack of information	PrEP resources lists	
Low PrEP awareness	HIV/PrEP information; PrEP eligibility assessment	
Low perceived risks	HIV risks assessment tool; HIV information/facts	
Concerns about PrEP use	PrEP related information (e.g., benefits, side-effects, cost, insurance policies)	
**System-level**		
Medical mistrust	Private AI-powered platform to assist non-judgmental and confidential conversation	
Confidentiality	Two-factor authentication platform for conversation	
Lack of navigation	PrEP navigation tools (e.g., PrEP clinics and care providers locators, and financial assistance program)	

## Position statement

The evolving development of behavioral intervention technologies provides opportunities to support users in changing behaviors and improving health. No conceptual model is available to guide technology-based behavioral interventions among vulnerable groups in the new era of digitalization. Our hybrid conceptual framework combines the Behavioral Model of Healthcare Utilization for Vulnerable Populations and the Integrated Model of Behavioral Prediction. Our integrated conceptual framework indicated that theoretical and technical components should be considered during a technological tool development to support the research community. A real-world intervention design has been demonstrated using the framework to guide behavioral intervention technologies to promote PrEP care and utilization among racial/ethnic minority women in the U.S. Using the integrated framework, researchers and practitioners can develop technology-assisted projects while incorporating key behaviors components for groups placed at increased risk.
